# Book Review: 17 Things Resilient Teachers Do (and 4 Things They Hardly Ever Do)

**DOI:** 10.3389/fpsyg.2021.702237

**Published:** 2021-05-25

**Authors:** Xiukui Ju, Ming Fan

**Affiliations:** ^1^Graduate School, Beijing Sport University, Beijing, China; ^2^School of Art, Beijing Sport University, Beijing, China

**Keywords:** resilient teachers, teacher-student relationship, student learning, education, stress management

The paramount role of the high-quality teaching profession and teacher effectiveness persistently elucidates that teachers' classroom practices have an undeniable impact on student learning and achievement. Furthermore, research has substantiated that how teachers feel about their personal lives and the extent to which they are fulfilled with the quality of their professional career can have thoroughgoing implications for their continuing professional development and their practices (Leithwood, 2018). Although there has been a surge of interest on teacher resilience, lively and engaging guidelines and hands-on strategies that explicate the significant role resilience plays remain scanty. Similarly, the question of how to prepare and nurture teachers to become great teachers to manage and tackle the challenges of the “uniquely demanding and emotionally taxing nature” (p. xi) of the teaching profession in an intellectual and emotional context of school and/or university has been contemplated for many years. The down-to-earth monograph, entitled *17 Things Resilient Teachers Do (And 4 Things They Hardly Ever Do)*, written eloquently by the bestselling author and educational consultant Bryan Harris, provides lucid answers to the abovementioned concerns. The prime objective of this volume is to help teachers develop, refine, sustain, and renew their capacities to manage stress and be finally resilient.

Structurally, following the introductory chapter, the book encompasses two parts, including 21 chapters. In the introductory chapter, the author succinctly conceptualizes teacher resiliency, justifies its indispensable role in teacher education, and encourages teachers to be resilient in order to be “the best teachers we can” (p. xi). In the rest of this chapter, the author delineates the structure of each chapter, mentioning that each chapter comprises three main sections. *In a Nutshell* section, a panoramic view of each chapter is illustrated; in the *Digging Deeper* section, insights, evidence, research, and anecdotes are presented; and in the most practical section, *Application Points*, specific strategies and techniques to build, nurture, and nourish resilience are provided.

Preceding the first chapter, the author revisits the definition of stress and its different types and argues rightly and cogently that the perception and control of stress are two central ideas that should be taken into account to be resilient. Put simply, the author stipulates that “if stress is the problem, resiliency is the answer” (p. 4). Moreover, Harris concludes his argument by stating that resiliency consists of *how* we think and *what* we do. In other words, it includes “our mental models, our mindsets, and our beliefs along with a specific set of coping mechanisms” (pp. 4–5).

Part I contains 17 chapters that thematically focus on what resilient teachers do. Space limitations preclude a detailed discussion of each individual chapter, so we give an overview of these 17 things that resilient teachers do. Commencing the first chapter with *Resilient Teachers Take Care of Their Health* puts a premium on the fact that “When you're healthy, the very best of you can be on display” (p. 6). Chapter 2, *Resilient Teachers Practice Gratitude*, emphasizes that expressing gratitude fosters resiliency by helping to mitigate the negative effects of the stressors around us. In order to achieve perspective and control and balance our thinking, we need to embark on framing, i.e., cognitive re-appraisal or positive self-talk, which constitutes the essence of Chapter 3, *Resilient Teachers Practice Framing*. In Chapter 4, Harris believes that *Resilient Teachers Understand the Power of “No”* by enumerating seven applicable points. Since “teaching is an emotionally taxing, tremendously rewarding roller coaster of a job” p. xi), *Resilient Teachers Manage Their Emotions* (Chapter 5).

As teachers, we need to be equipped with some strategies to relieve our stress, so Chapter 7 presents these tools as *Resilient Teacher Practice In-The-Moment Stress Relievers*. Chapter 8, *Resilient Teachers Develop a Professional Support Network*, argues that possessing an amicable network of friends, colleagues, and mentors improves job satisfaction, strengthens our immune system, and assists us in managing stress. “Remembering our faith tradition, attending to ordinary things and giving attention to passions and interests not related to the profession” (p. 44) entail that *Resilient Teachers Have a Life Outside the Classroom*. Because being disorganized makes us stressed out, Chapter 10 accentuates that *Resilient Teachers Get Themselves Organized*.

*Resilient Teachers Focus on What They Can Control* (Chapter 11) and *Resilient Teachers Know How to Receive Feedback* (Chapter 12) are two other important tools that can help us bounce back and navigate difficult waters in our teaching profession. Another pivotal resiliency strategy is encapsulated in Chapter 13, *Resilient Teachers Advocate for Themselves*. The author opines that self-advocacy and self-efficacy are two other ingredients of successful resilient teachers. Since setting goals empowers teachers, Chapter 14 echoes that *Resilient Teachers Create and Track Goals*. The other three chapters in Part I deal with *Resilient Teachers Unplug* (Chapter 15), *Resilient Teachers Laugh and Have Fun with Their Students* (Chapter 16) and *Resilient Teachers Help Students Build Resiliency* (Chapter 17). What has intrigued us the most as the reviewers is that the strategy in Chapter 17 strengthens the teacher-student relationship, which makes the cornerstone of the teaching and learning process.

Focusing on the four things resilient teachers hardly ever do is the focus of Part II, which comprises the remaining four chapters. The author convincingly argues that resilient teachers hardly ever beat themselves up over past mistakes, spend much time complaining, freak out about the change, and shy away from conflict.

All in all, we believe that this insightful and thought-provoking monograph invites us to explore deeper into the dynamics and complexities of teacher resiliency and reminds us that nurturing and nourishing the capacity for resilience is more than an individual responsibility. Hence, we fully endorse this user-friendly contribution since this opportune volume cultivates healthy individual and collective learning opportunities which help teachers live a *resilient* and *stress-free* profession.

## Author Contributions

XJ took the lead in writing the manuscript. All authors provided critical feedback and helped shape the research.

## Conflict of Interest

The authors declare that the research was conducted in the absence of any commercial or financial relationships that could be construed as a potential conflict of interest.
